# *Aralia cordata* Thunb. as a Source of Bioactive Compounds: Phytochemical Composition and Antioxidant Activity

**DOI:** 10.3390/plants11131704

**Published:** 2022-06-28

**Authors:** Viktorija Puzerytė, Pranas Viškelis, Aistė Balčiūnaitienė, Paulina Štreimikytė, Jonas Viškelis, Dalia Urbonavičienė

**Affiliations:** Lithuanian Research Centre for Agriculture and Forestry, Institute of Horticulture, 54333 Babtai, Lithuania; viktorija.puzeryte@lammc.lt (V.P.); pranas.viskelis@lammc.lt (P.V.); paulina.streimikyte@lammc.lt (P.Š.); jonas.viskelis@lammc.lt (J.V.); dalia.urbonaviciene@lammc.lt (D.U.)

**Keywords:** *Aralia cordata*, plant morphological parts, bioactive compounds, antioxidant activity, DPPH^•^, ABTS^•+^, FRAP

## Abstract

Plant primary and secondary metabolites are a significant source for many applications, including extractions of functional components, green synthesis development, and producing higher-added value products. However, in the variety of botanicals, *Aralia cordata* Thunb. plant is getting attention for its similarity to ginseng. This study comprehensively examines the biochemical and phytochemical profiles of different *A. cordata* morphological parts: root, stem, leaf, inflorescence, berry, and seed. Additionally, the establishment of total phenolic content and quantitative analysis of powerful antioxidants such as chlorophyll, carotenoids (zeaxanthin, lutein, and β-carotene), proanthocyanidins, and anthocyanins content were evaluated. The results indicated that *A. cordata* stem and berries are an excellent source of anthocyanins in the range from 18.27 to 78.54 mg/100 g DW. Meanwhile, the antioxidant activity was evaluated using three different methods based on the capacity to scavenge: DPPH^•^ scavenging capacity, ABTS^•+^ radical cation assay, and ferric reducing antioxidant power (FRAP) and ranged from 27 to 168 µmol TE/g DW, 8 to 117 µmol TE/g DW, and 18 to 157 µmol TE/g DW, respectively. This study proposes a novel competitive plant for many health-promoting applications in the nutraceutical, pharmaceutical, material, and food industries.

## 1. Introduction

These days there has been more interest in plants for medicinal and therapeutic purposes against various diseases and disorders and health benefits [1]. Phytochemicals and secondary metabolites are the main drivers of medicinal plants for medicinal action [2]. *Panax ginseng* is a popular medicinal herb, and there has been a worldwide interest in plants with similar bio stimulatory properties as ginseng (*Panax ginseng*). For centuries legendary ginseng plant was irreplaceable; however, after lengthy studies of plants, botanists discovered that *Panax ginseng* and *Aralia cordata* Thunb. (*A. cordata*) belong to the family Araliaceae [3]. *A. cordata* is a powerful medical herb native to Asia [4,5].

Plants possess many molecules that exhibit significant antioxidant activity and can prevent oxidative damage of different cellular components [6]. Phenolic compounds among these molecules are presented as one of the primary sources of the antioxidant activities displayed, which can scavenge free radicals, quenching singlet and triplet oxygen, or decomposing peroxides [7]. In addition, it is reported that phenolic compounds have anticancer, antibacterial, and anti-inflammatory activities [8,9]. Besides phenolic compounds, carotenoids, tocopherols, and ascorbic acid are natural antioxidants [10]. Chemical materials in plants, such as carbohydrates, protein, vitamins and fibre also contribute to the antioxidant capacity [11]. The most common in vitro methods used to establish the antioxidant activity are ABTS^•+^ (2,2′-azinobis(3-ethylbenzothiazoline-6-sulphonic acid)) free radical scavenging activity assay, DPPH^•^ (1,1-diphenyl-2-picrylhydrazyl) free radical scavenging activity assay and ferric ion reducing antioxidant power assay (FRAP).

In this study, the different morphological parts of the *A. cordata* used in traditional medicine were analysed to evaluate the existence of preferential bioactive compounds. Furthermore, several studies are available in the literature that provide data on the chemical composition of some parts of *A. cordata* [12,13,14].

The root of the *A. cordata* plant is used for lumbago, rheumatism, and lameness in traditional Chinese medicine [4]. Furthermore, one research reports that *A. cordata* root methanol extract might help to prevent lipid metabolism disorders and their complications [15]. However, only diterpenes and essential oils are detected in the root, which show analgesic, anti-inflammatory, ref. [5,16] and cytotoxicity activities [17]. Some research discussed the isolation of diterpenes, polyacetylenes, saponins, lipid glycerol, and sterols from *A. cordata* roots [18,19,20]. Literature analysis revealed that the phenolic compounds, nucleotides, saccharides, and alkaloids are isolated only from the roots [14]. Moreover, promising research was done about polyacetylene and araliadiol. These compounds were isolated from *A. cordata* ethanolic extracts. Araliadiol showed potent cytotoxicity before human breast carcinoma cells (MCF-7) [21]. In addition, the study of leaf and stem extracts reported that diterpenes, triterpenes, and saponins were isolated as active inhibitory constituents against COX-1 and COX-2 [22]. Furthermore, *A. cordata* leaf and stem extracts showed a strong anti-inflammatory effect [23], and in experimental animal models, therapeutically activity was seen to prevent dementia and stroke [24]. 

**Figure 1:**
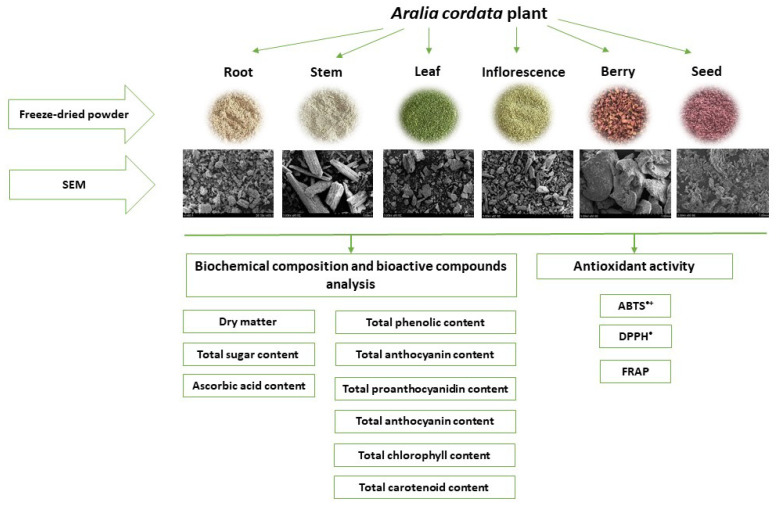
Overview of the graphical experimental design for *A. cordata* morphological parts evaluation.

The study aimed to determine the bioactive compounds and antioxidant capacity of different parts of *A. cordata*. This paper presents a comprehensive study of *A. cordata* root, stem, leaf, inflorescence, berry, and seed investigation of the biochemical composition, the bioactive compounds (total phenolic compounds, anthocyanins, proanthocyanidins, chlorophylls, carotenoids), and the antioxidant capacity. The graphical experimental design of *A. cordata* morphological parts evaluation is presented in Figure 1. Furthermore, scanning electron microscopy (SEM) images of the plant parts were used to analyse modifications in particle shapes after freeze-drying and milling. 

## 2. Results and Discussion

### 2.1. Determination of Sugars, Sucrose, Ascorbic Acid and Dry Matter Content

The composition of sucrose, inverted sugar, total sugar content, and ascorbic acid of *A. cordata* root, stem, leaf, inflorescence, berry, and seed is presented in Table 1. The highest dry matter amount was detected in stem (97.6%), whereas the lowest in berry (90.9%). The most significant amount of total sugars was determined in berry (50.8%), of which inverted sugar stood at 44.2% and sucrose at 6.5%. The lowest total sugar content was detected in the root (5.7%), 89% lower than it was obtained in berries (Table 1). Ganguly et al. (2018) [25] reported that the total sugars of Indian ginseng (*Withania somnifera*) are 7.17%, which is similar to the obtained results. 

The amount of ascorbic acid varied from 22.3 to 146.0 mg/100 g DW in *A. cordata* morphological parts. The amount of ascorbic acid in stem, leaf, and inflorescence was similar (66.4–67.2 mg/100 g DW). Meanwhile, Han et al. [26] obtained ascorbic acid (47.00 mg/100 g DW) content of *Aralia continentalis* leaf subspecies of *A. cordata*, slightly lower than our obtained results. The fewest ascorbic acid was obtained in the root (22.3%), 3 times lower comparing with stem, leaf, and inflorescence. On the contrary, the greatest amount of ascorbic acid was obtained in the berry (146.0 mg/100 g DW). The determined results are slightly higher than was obtained in *A. cordata* botanical relative *Oplopanax horridum* berries 30.3% [27].

### 2.2. Evaluation of Chlorophyll, Phenolics, Carotenoids, Proanthocyanidins and Anthocyanyn Contents of A. cordata Morphological Parts

Chlorophyll is a powerful pigment that gives green color to leaves and decisive in photosynthesis capacity and indirectly for the primary compounds that are the basis for synthesizing secondary metabolites [28,29]. Due to various biological effects such as antigenotoxicity and antioxidant activity, chlorophylls have been recommended as essential dietary agents [30]. Chlorophylls can inhibite mitochondrial radical species, have antiproliferative effects on cancer cells, and regulate the redox capacity [31]. The result of chlorophyll pigment content shows that the greatest content of total chlorophyll (7.45 mg/g DW) and chlorophyll a and b was detected in leaves (Table 2). In contrast, total chlorophyll was not detected in the root and was not investigated in berry, and seed. Chlorophyll a and b is similar but different in color. Chlorophyll a is bluish-green and chlorophyll b is yellow-green. Based on findings from the literature, chlorophyll content and greenness depend on light and temperature [32,33].

Phenolic compounds are essential components with antioxidant properties in plants [34]. Significantly, various environmental and abiotic factors, including seasonal variations, water content, temperature, growth conditions, and light, greatly influence variation in the collection and composition of phytochemicals in plants [35]. The result of total phenolic content is presented in Table 3. The amount of total phenolic content ranged from 333.79 to 2328.31 mg GAE/100 g DW. The most significant value was detected in leaf 70% aqueous ethanol extract (2328.31 mg GAE/100 g DW), which is 86% higher than it was detected in the lowest value in the stem extract (Table 3). It can be seen that the amount of total phenolic content of *A. cordata* morphological characteristics decreases in the following order: leaf > inflorescence > berry > root > seed > stem extracted with 70% aqueous ethanol extract. Furthermore, there is a significant difference (*p* < 0.05) in total phenolic content among all *A. cordata* parts. In comparison, Zaluski et al. (2012) analyzed five *Eleutherococcus* species and determined the total phenolic content in 75% ethanolic extracts ranging in roots from 690 to 1060 mg GAE/100 g DW, in leaves from 2030 to 3720 mg GAE/100 g DW and in fruits from 610 to 1970 mg GAE/100 g DW [36]. The results are similar to our investigated total phenolic results of *A. cordata* morphological parts. Meanwhile, Qi et al. (2018) analyzed *Aralia elata* buds collected from eight geographic regions of China and determined that the total phenolic content varied from 285 to 1455 mg/100 g [37]. In addition, we compared the results with *A. cordata* botanical relative *Panax ginseng*, which may have a strong correlation between plants’ phenolic content. Jung et al. (2006) revealed that the total phenolic content of ginseng leaf ethanol extract is 2333 mg/100 g DW, which is similar to our investigated *A. cordata* leaf 70% aqueous ethanol extract results (2328 mg/100 g DW) [38]. Phenolic compounds such as phenolic acids, flavonoids, and tannins contain various biological activities, for instance, anti-inflammatory and anti-carcinogenic [39]. Accordingly, high phenolic content is crucial in establishing the antioxidant activity of plant morphological parts. 

Anthocyanin pigments are responsible for blue, red, and purple colors in plant berries, fruits, and stems [40]. Anthocyanins are flavonoid phytopigments [41], known as strong antioxidants with extremely high radical scavenging activities [42]. Their usage has been related to positive reductions in oxidative stress and inflammation in humans [43]. Recent interest in anthocyanin pigments has intensified due to potential health improvements such as the lower risk of coronary heart disease, anti-carcinogen activity, stroke, anti-inflammatory impacts, increased visual acuity, and enriching cognitive behavior [44,45]. Based on the research, the anthocyanin content varied from 18.27 to 78.54 mg/100 g DW in samples. The most significant content of anthocyanin pigment and proanthocyanidins was detected in the berry at 78.54 mg/100 g DW (Table 3). Meanwhile, Tian et al. (2021) analyzed *Aralia elata* fruit selected from five different regions (Jiaohe, Harbin, Raohe, Benxi, and Mudanjiang) in Northeast China and determined that the anthocyanin content varied from 65 to 270 mg/100 g [46]. Moreover, Chen et al. (2020) investigated *A. cordata* botanical relative *Acanthopanax sessiliflorus* fruits and obtained anthocyanin content (121.35 mg/100 g fw) [47].

In plants, proanthocyanidins are oligo- and polymeric end products of the flavonoid biosynthesis pathway and are also known as condensed tannins [48]. Proanthocyanidins possess various therapeutic properties and act as an antioxidant; therefore, they exhibit different pharmacological properties such as anticancerous, antiallergic and anti-inflammatory, antimicrobial, antiviral, healing of wounds [49,50]. Proanthocyanidin content results show that the most significant value was detected in berries 0.28 mg/100 g DW (Table 3). Therefore, based on the results obtained, *A. cordata* cannot be recommended as a valuable source of proanthocyanidins.

Carotenoids are responsible for the yellow, red, and orange colors found in nature. The color of carotenoid is masked in green plant tissues by the more dominant pigment chlorophyll and becomes visible during the degradation of chlorophyll [51,52]. According to their function, carotenoids are separated into two groups: carotenes, which include lycopene, α-carotene, and β-carotene, and xanthophylls, which contain zeaxanthin and lutein [53]. Humans and animals cannot synthesize them in vivo, and can be obtained only through diet [54]. Carotenoids have various biological functions that contribute to therapeutic effects, including anticancer, immunomodulatory, anti-inflammatory, antibacterial, antidiabetic, and neuroprotective [55,56]. Furthermore, carotenoids reduce oxidative stress in the host body by acting as a free radical scavenger and upregulating the production of antioxidant enzymes [57,58]. The result of carotenoid content shows that the most significant value of carotenoids (0.812 mg/g DW) pigment was obtained in the leaves (Table 4). Meanwhile, Belin et al. (2021) analyzed *Aralia nudicaulis* total carotenoid content of aboveground (0.73 mg/g DW) and belowground (0.03 mg/g DW) parts, and it is slightly lower than it was obtained in our results [59]. Meanwhile, Chanwitheesuk et al. (2005) reported that the total carotenoid content of three selected Araliaceae family medical herbs leaves varied from 2.52 to 3.83 mg/100 g DW. A greater content was obtained in *Macropanax dispermus* (3.83 mg/100 g DW), whereas lower content was determined in *Polycia fruticosa* (2.52 mg/100 g DW) [59]. However, the results presented are lower than those detected in *A. cordata* leaves. 

HPLC–DAD quantitative analysis of carotenoid extract was used to identify individual carotenoids present in *A. cordata* different morphological parts expressed in percentage concentration of total carotenoid content (of total Car content). The carotenoids were studied by comparing the UV–VIS spectra and retention time of sample peaks with those of the standards. As a result, three compounds were identified in the stem, leaf, inflorescence, berry, and seed: lutein, zeaxanthin, and β-carotene (Table 4). Lutein was identified as the primary compound in leaf, inflorescence, and stem (Table 4), but the concentration varied significantly (*p* < 0.05) between plant parts. The lowest amount of this pigment was observed in the berry (0.012 mg/g DW) and seed (0.011 mg/g DW) samples, and it was even higher in inflorescence (0.271 mg/g DW) and leaf (0.291 mg/g DW). Contrary, Othman et al. (2017) reported that the lutein content of 20 selected medical herb leaves ranged from 0.116 to 0.180 mg/g DW, from which the highest value was detected in *Pogostemon cablin* leaves [60]. According to research reports, lutein may decrease age-related disorders due to its antioxidant and anti-inflammatory properties [61]. The quantity of zeaxanthin observed was 24.0 and 26.1% (of total Car content) in leaf and inflorescence, respectively, and 33.1 and 29.4% (of total Car content) in stem and seed. Meanwhile, the most significant quantity of β-carotene was observed in the leaf (Table 4), and the lowest concentration was detected in the seed. The zeaxanthin concentration in berries was not calculated because it was within the limit of quantitation. 

### 2.3. Determination Antioxidant Capacity of A. cordata Morphological Parts

**Figure 2:**
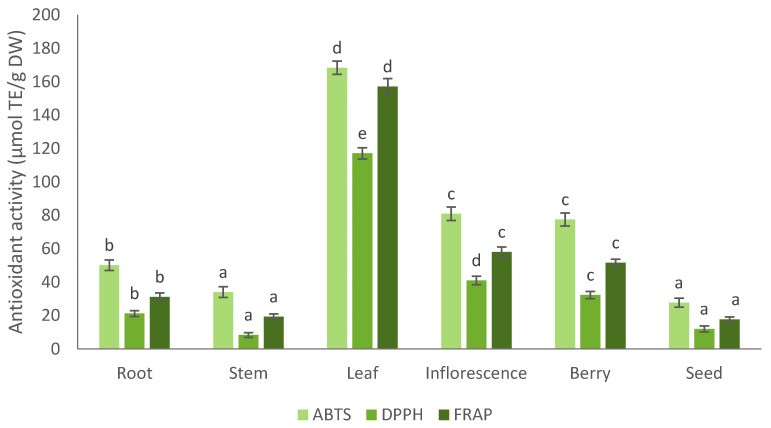
The antioxidant activity of *A. cordata* different morphological parts evaluated by using ABTS, DPPH, FRAP assays. Values were expressed as mean ± standard deviation (*n* = 3); different letters indicate statistically significant differences between plant parts (one-way ANOVA and Tukey’s HSD test, *p* < 0.05).

Antioxidants have shown the ability to decrease or end the progression of several chronic diseases [62]. Plant parts extracts are natural multicomponent matrices comprising a variety of biologically active compounds, each of which has its own mechanism of antioxidant activity. Wherefore, antioxidant capacity of plant extracts cannot be suitably tested using only one methodology [63,64]. For these reasons, to analyse the antioxidant capacity of plant extracts, it is suggested to use more than one method [65]. In order to properly evaluate the antioxidant capacity of the ethanol extracts of *A. cordata* morphological parts, different antioxidant activity assays (ABTS^•+^, DPPH^•^ and FRAP) were applied. The antioxidant capacities were analysed with an ABTS^•+^ radical cation decolorization assay, the DPPH^•^ radical scavenging method, and the ferric reducing antioxidant power (FRAP) assay. The ABTS^•+^ antioxidant capacity values of *A. cordata* morphological parts extracts range from 27 to 168 µmol TE/g DW (Figure 2). The highest antioxidant activity was obtained in the leaf 70% aqueous ethanol extract, which is 84% higher than it was detected in the lowest value in the seed extract. *A. cordata* stem and seed extracts, as well as inflorescence, and berry extract did not show significant difference (*p* < 0.05), respectively.

The DPPH^•^ antioxidant capacity values of *A. cordata* morphological parts extracts range from 8 to 117 µmol TE/g DW (Figure 2). The greatest antioxidant activity was determined in the leaf 70% aqueous ethanol extract, which is 93% higher than it was detected in the lowest value in the stem extract. On the other hand, *A. cordata* stem, seed and root extracts as well as inflorescence and berry did not show significant difference. 

*A. cordata* root, stem, leaf, inflorescence, berry, and seed extracts results of ferric reducing antioxidant power (FRAP) assay are presented in Figure 2. The FRAP antioxidant activity values of *A. cordata* morphological parts extracts range from 18 to 157 µmol TE/g DW. The most significant antioxidant activity was established in the leaf 70% aqueous ethanol extract whereas the lowest was obtained in the seed extract, which is 89% lower than the highest value. *A. cordata* stem and seed, as well as inflorescence and berry extracts, did not show significant differences, respectively. 

The results in all radical scavenging assays demonstrated that the greatest value was obtained in leaf 70% aqueous ethanol extract of *A. cordata*. On the contrary, the lowest value varied between the stem and seed extracts, and it was lower than 84% in all assays (Figure 1). The antioxidant activity values difference between *A. cordata* parts of the plant can be explained by the accumulation of secondary metabolites, which depend on photosynthesis and environmental factors such as seasonality, sunlight, temperature, and humidity [66]. A significant connection was revealed between the total phenolic compound content and antioxidant capacities. Our research showed a similar antioxidant activity correlation as the previously determined in the different morphological parts of ginseng [67]. Ryu et al. (2020) reported that ginseng leaves extracts have the highest antioxidant activity comparing to flower and berry extract at the DPPH^•^, ABTS^•+^ antioxidant activity assays [67]. The highest total phenolic contents of the leafs extract compared with the root, stem, berry and seed extracts might explain the significant antioxidant activities of the leaves. These results indicate that *A. cordata* leaves are adequate to be a potential source of antioxidants or functional food as an alternative to synthetic compounds.

### 2.4. Evaluation of Colour Changes in A. cordata Different Morphological Parts

CIEL*a*b* color coordinates of all morphological parts of *A. cordata* (root, stem, leaf, inflorescence, berry, seed) were measured to evaluate the possible use of different morphological parts for the development and production of various functional foods and pharmaceutical ingredients [68]. Color is a significant physical indicator that complements the microstructure and morphology of the different plant parts. Color also depends on the drying technique. Therefore, freeze-drying may be a considerable technique to preserve the plant color, vitamins, antioxidant activity, and bioactive compounds [69]. Plant parts showed an intensive color after freeze-drying and milling, which would be acceptable for an application in the food industry as a colorant (Table 5).

Brightness has a significant difference between *A. cordata* morphological parts, stem showed the highest brightness L* values (72.1), whereas the lowest L* value was detected in the berry (36.7). The values of greenness (negative a*) showed stem, leaf, and inflorescence, though redness (positive a*) value was found in the root, berry, and seed. The lowest b* value was detected in the berry (3.5) (Table 5). The highest C value was found in the inflorescence, which is 71% higher than the lowest value. The higher value of C, the higher the color purity (saturation). Hue angle h° showed a significant difference between the investigated *A. cordata* morphological parts, which shows the color applicability of the various parts of this plant. These color indicators are important in the food industry as they influence the potential product’s color characteristics and the consumer’s choice.

### 2.5. Principal Component Analysis (PCA) of A. cordata Morphological Parts

**Figure 3:**
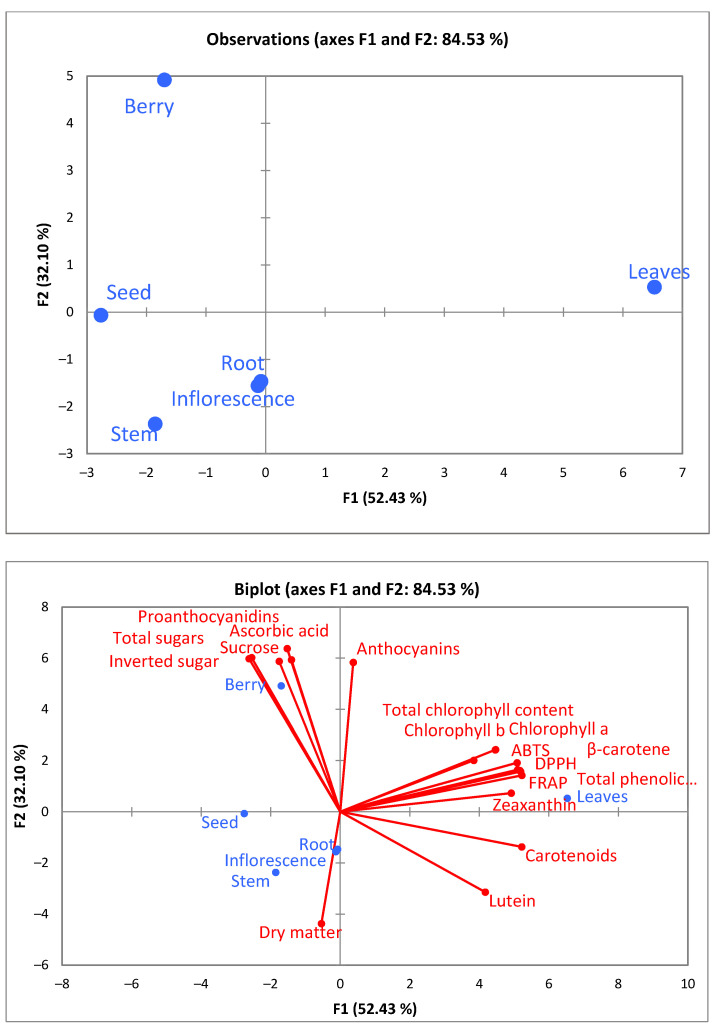
Principal component analysis (PCA) shows how different morphological parts differ from each other by their biochemical composition.

A principal component analysis (PCA) provides a map of how the different morphological parts relate to each other according to an obtained analysis of the bioactive compounds, antioxidant activity, biochemical composition, and individual carotenoids. The score plot models of investigated morphological parts are shown in Figure 3.

The PCA’s first two factors (F1 vs. F2) explained 84.53% of the total data variance, as demonstrated in the scatterplot (Figure 3). F1 explained 52.43%, whereas F2 explained 32.10% of the total variability. According to the PCA analysis, it can be seen that the leaves have the greatest content of total chlorophyll and chlorophyll a and b, β-carotene, and zeaxanthin. Moreover, leaves have a significantly high total phenolic content and antioxidant effect in all antioxidant capacity assays. Meanwhile, the berries are rich in proanthocyanidins, anthocyanins, ascorbic acid, and total sugar. Therefore, it is clear that morphological parts are rich in bioactive compounds, which may have health benefits. Furthermore, these data are helpful in zero-waste processing and the circular economy, where all parts of a plant can be used for multiple purposes. Additionally, it is reported that leaves (23%) are the most widely used plant part, followed by the whole plant (15%), aerial parts (12%), fruits (10%), seeds (7%), rhizomes (6%) and the other parts (stems, roots, bark, flowers, and twig) are presented lower than 6% [70].

### 2.6. Scanning Electron Microscopy (SEM) Analysis

**Figure 4:**
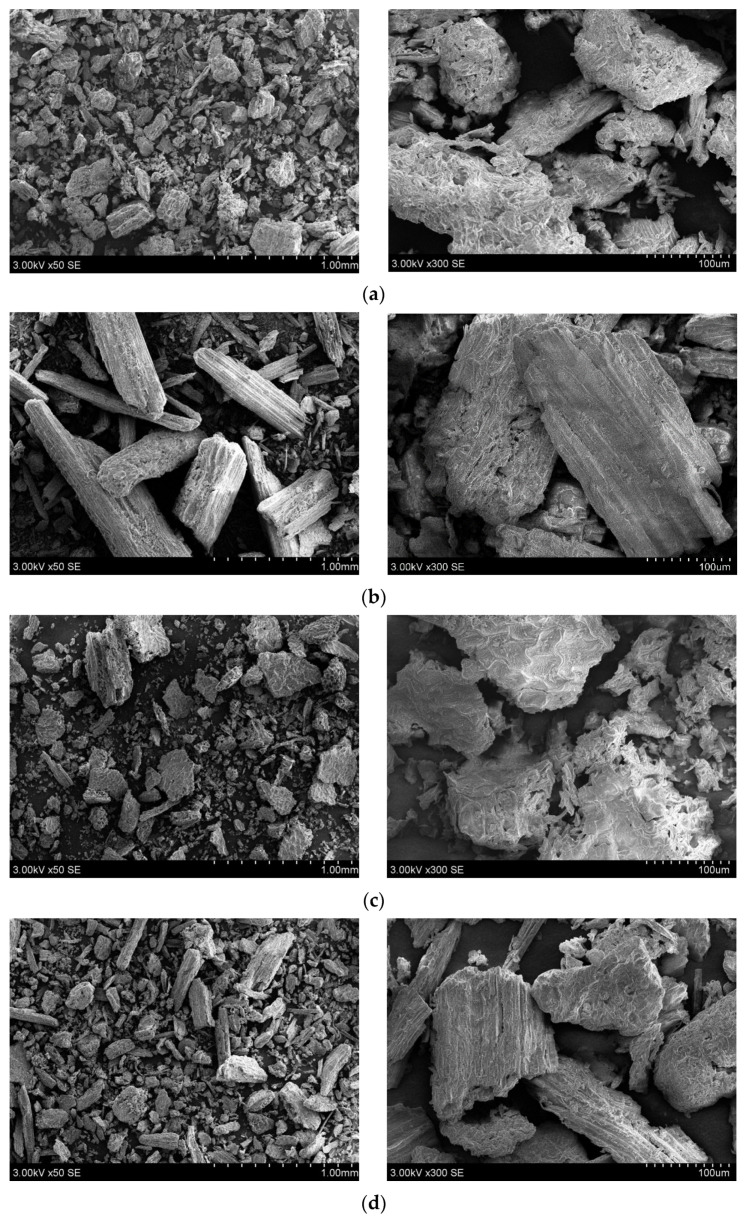

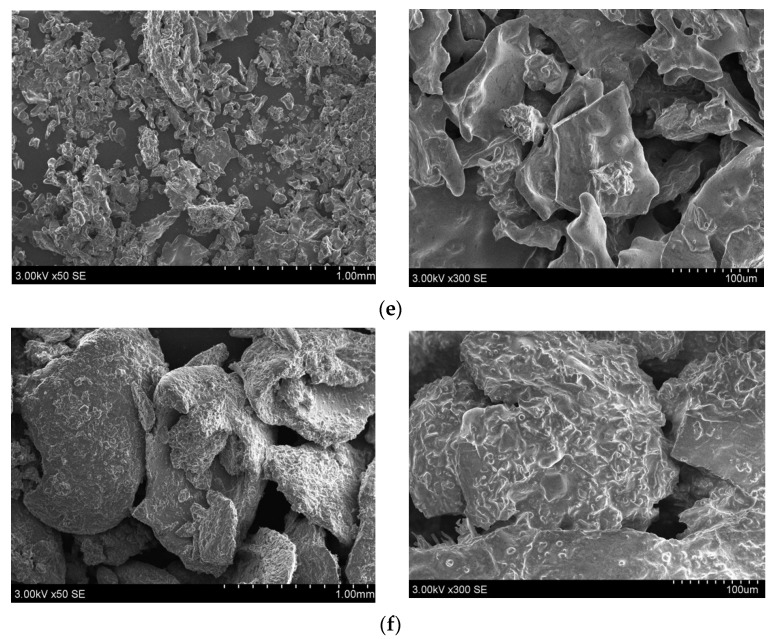
SEM morphology images of *A. cordata* freeze-dried samples powders (**a**) root, (**b**) stem, (**c**) leaf, (**d**) inflorescence, (**e**) berry, (**f**) seed.

The microstructure and morphology of the different plant parts were investigated using a scanning electron microscope (SEM) and the results show differences between the *A. cordata* morphological parts. SEM analysis was applied to visualize the surface morphology and particle size of *A. cordata* plant. The results of the analysis are presented in Figure 4.

SEM photos of *A. cordata* root, stem, leaf, inflorescence, berry, and seed freeze-dried powders presented in Figure 4, show that particles are irregular geometric shape, the surface is rough and layered. It is possible that the mechanical processing such as freeze-drying and milling had a strong impact on a particle form and size [71]. Tested plant root particle surface has greater roughness. Moreover, microfibrils and amorphous zones can be seen on the surface (Figure 4a). As (Figure 4b) showes *A. cordata* stem particles have oblong shape and do not form agglomerates. Moreover, the detected chips and porosity are visible on the surfaces of the particles. Leaf and inflorescence freeze-dried powder SEM pictures are presented in Figure 4c,d, respectively. Particles share similar geometric form and are evident flake in shapes. SEM photos of berry powder show that the particles are much smaller in size, tending to form agglomerates (Figure 4e) because of their hydrophilic nature. Materials of cellulosic origin can explain high hydrophilicity with hydroxyl groups in the macromolecule. The morphology of *A. cordata* depends on the individual morphological parts of the plant. For example, there are particles of different sizes with different crystalline and amorphous zones. The SEM photos of *A. cordata* freeze-dried seed powder presented in Figure 4f present derivatives of various sizes and irregular shapes. 

According to the scientific literature, it was necessary to investigate the morphology of the plant, because, the amount of bioactive substances may also depend on the morphology such as particles’ size and volume ratio [72]. The use of *A. cordata* in traditional foods may improve the product’s functional properties. In addition, increasing the efficiency of the industrial processing of this plant could be found in direct application in the food, pharmaceutical, and cosmetic industry. Furthermore, sustainably alternative plant-based materials can be used as a filler to polymer materials, thus creating a secondary or tertiary use of the materials, such as polymer composite [73]. Such polymeric composites with plant-based fillers are sustainable, environmentally friendly, biodegradable and reduce the use of plastics. However, their topology, morphology, surface texture and roughness must be analysed before this can be done. These parameters are important due to the dispersibility in the polymer matrix and the adhesion between the filler particles and the polymer. For these reasons, it is essential to study the morphology of the particles properly.

## 3. Materials and Methods

### 3.1. Chemicals

The following substances and solvents were used in the study: ethanol 96% (*v/v*) (AB Strumbras, Kaunas, Lithuania), Folin–Ciocalteu reagent, gallic acid (3,4,5-trihydroxybenzoic acid, 99%), ABTS^•+^ (2,20-azino-bis(3-ethylbenzothiazoline-6-sulfonic acid), NaCl, KH_2_PO_4_, Na_2_HPO_4_, KCl, K_2_S_2_O_8_, DPPH^•^ (2,2-diphenyl-1-picrylhydrazyl hydrate free radical), Trolox (6-hydroxy-2,5,7,8-tetramethyl-chroman-2-carboxylic acid), Na_2_CO_3,_ potassium acetate, acetic acid, TPTZ (2,4,6-Tris(2-pyridyl)-s-triazine) (Carl Roth, Karlsruhe, Germany), iron (III) chloride hexahydrate (Vaseline-Fabrik Rhenania, Bonn, Germany), DMCA (4-(dimethylamino)-cinnamaldehyde).

### 3.2. Plant Material 

*A. cordata* plant was grown at the Institute of Horticulture, Lithuanian Research Centre of Agriculture and Forestry experimental fields (55°08‘ N, 23°80‘ E). The *A. cordata* in the experimental field was planted in the spring of 2016. *A. cordata* sprouts were obtained from the farmer Roman Kuprys (Dotnuva, Lithuania, 55°35‘ N, 23°91‘ N). The planting distance was 1 × 1 × 1 m. Soil type—*Calcic Endogleyic Luvisol* (*LV-gl-n-cc*), pH—7.1, humus—2.8%, P_2_O_5_—255 mg kg^−1^, K_2_O—230 mg kg^−1^, average annual precipitation—630 mm, the average sum of active temperatures (>10 °C)—2300°. *A. cordata* was harvested in 2020. Samples of *A. cordata* different morphological parts were randomly collected at the stages of flowering (phenological development stage (BBCH) 65), fruiting (BBCH 88), and root (BBCH 99) [74]. The collected plants were separated into different morphological parts (root, stem, leaf, inflorescence, berry, and seed) and instantly frozen at −35 °C before freeze-drying. The samples were lyophilized in Zirbus lyophilizer (Zirbus Technology GmbH, Bad Grund, Germany) at 0.01 mbar pressure and −85 °C condenser temperature. The freeze-dried different morphological parts were grounded to a powder using a Retsch 200 knife mill (Haan, Germany) and stored in a sealed container, prior to the analysis.

### 3.3. Extraction

To analyse phenolic compounds and antioxidant activity, 2 g of morphological plant powder was weighted, and 50 mL of 70% (*v/v*) ethanol was added. For the analysis of anthocyanidin and proanthocyanidins, 2 g *A. cordata* powder was weighted, and 50 mL of 70% (*v/v*) ethanol acidified with 0.1 M HCl was added. The extraction process continued for 24 h in the dark. Following the extraction, the samples were centrifuged for 5 min at 8500 rpm in a Biofuge Stratos centrifuge and established supernatants were filtered through a membrane filter with a pore size of 0.22 μm (Carl Roth GmbH, Karlsruhe, Germany). For the chlorophylls and carotenoids analysis, 500 mg of *A. cordata* plant samples were moved to a ceramic pestle. The pigments were extracted and transferred to a volumetric flask (100 mL) with an 80% acetone/water mixture. The homogenised sample mixture was centrifuged at 10,000 rpm for 15 min at 4 °C. The supernatant was separated and instantly subjected to analysis. 

### 3.4. The Determination of Dry Matter Content

The dry matter content was established of freeze-dried samples powder, and it was drying in a Universal Oven ULE 500 at 105 °C (Memmert GmbH + Co. KG, Schwabach, Germany) at a constant weight. 

### 3.5. The Determination of Ascorbic Acid

The quantity of ascorbic acid was measured by official titrimetric method [75].

### 3.6. The Evaluation of Sugars

The amounts of sucrose, monosaccharides, and total sugars were obtained according to the Association of Official Analytical Chemists [76].

### 3.7. Measurment of Chlorophyll a and Chlorophyl b Content

The total chlorophylls, chlorophyll a, and chlorophyll b content were analysed spectrophotometrically, with the method of Lichtenthaler and Buschmann [77] as described in [78]. Chlorophyll a and chlorophyll b were determined spectrophotometrically. The absorption was measured with a Cintra 202 spectrophotometer (GBC Scientific Equipment Pty Ltd., Braeside, VIC, Australia), and the results were investigated with the Cintral ver.2.2 program.

### 3.8. Analysis of Carotenoids

The content of total and individual carotenoids in *A. cordata* different morphological parts was analysed with HPLC. The content of individual carotenoids was expressed as percentage concentration of total carotenoids content. For HPLC analysis, aqueous 80% acetone extracts previously filtered through PVDF membrane filters with a pore size of 0.45 μm (Millipore, Burlington, MA, USA) and analysed Chromatographic separation was performed using Waters HPLC system consisting of 2695 liquid separation module, UV–vis detector 2489 (Waters Corporation, Milford, MA, USA). Chromatographic separations were carried out by using a RP-C_30_ column, (5 µm, 4.6 × 250 mm, YMCTM Europe, Dinslaken, Germany) and a C_30_ guard column (5 µm, 10 × 4.0 mm, YMC Europe, Dinslaken, Germany). Qualitative and quantitative analysis of carotenoids compounds were performed according to the previously described method Bobinaitė et al. (2020) with slight modifications [79]. The column temperature is 22 °C. The individual carotenoids were detected at 450 nm. The mobile phase consisted of methyl-*tert*-butyl ether (solvent A) and methanol (solvent B). The flow rate was 0.6 mL/min. The samples were injected at 99% B (held 1 min), and the gradient then changed to 0% B (90 min) and again to 99% B in 5 min (held 5 min). The calibration curves were generated by linear regression analysis using authentic lutein, zeaxanthin, all-*trans*-β-carotene standard (concentration range was from 0.01 to 5.0 mg/100 mL). The coefficient of determination (R^2^) of the calibration curve was 0.099–0.996. The limit of detection (LOD) was 0.015–0.035 mM and the limit of quantitation (LOQ) was 0.022–0.067 mM.

### 3.9. Determination of Total Proanthocyanidin Content

Total proanthocyanidin content was established with the DMAC method [80]. 20 μL of the diluted extracts were mixed with 3 mL DMAC solution. The absorption was measured after 5 min with Shimadzu UV-1800 spectrophotometer at 640 nm against reagent blank. A triplicate determination was made from each extract. Epicatechin (0.002–0.016 mg/mL) was used as a standard. The results in the extracts expressed as epicatechin equivalents per dry weight (mg/100 g DW).

### 3.10. Determination of Total Anthocyanin Content 

Total anthocyanin content was established with pH difference method [81] spectrophotometrically. The absorbance of the samples was measured at 520 nm and 700 nm and determined as cyanidin-3-*O*-glucoside equivalent. The result of anthocyanins was determined from a calibration curve and expressed as mg/100 cyanidin-3-*O*-glucoside per gram of dry weight (mg/100 g DW).

### 3.11. Determination of Total Phenolic Content 

Total phenolic content was determined by the Folin–Ciocalteu’s method [82] using gallic acid as the standard. The absorbance of the samples was measured at 765 nm using a Cintra 202 (GBC Scientific Equipment, Knx, Braeside, VIC, Australia) spectrophotometer. The total phenolic content was calculated from a gallic calibration curve and expressed as mg/100 gallic acid equivalent (GAE) per gram of dry weight (mg GAE/100 g DW).

### 3.12. Determination of Antioxidant Capacity

An ABTS^•+^ radical cation decolorization assay was adjusted according to the methodology described by Re and colleagues [83] with some modifications. A volume of 2 mL of ABTS^•+^ (2,2’-azino-bis(3-ethylbenzthiazoline-6-sulphonic acid)) solution (absorbance 0.800 ± 0.02) was mixed with 20 μL of samples. The absorbance decreasing of each sample was measured at 734 nm in a Cintra 202 (GBC Scientific Equipment, Knox, Braeside, VIC, Australia) spectrophotometer after 30 min.

The DPPH^•^ free radical scavenging activity was established using the method suggested by Brand Williams, Cuvelie and Berset [84] with some modifications [85]. 2 mL DPPH^•^ (2,2-diphenyl-1-picrylhydrazyl) solution in 99.0% *v/v* ethanol was mixed with 20 μL of samples. A decrease in absorbance was determined at 515 nm in a Cintra 202 (GBC Scientific Equipment, Knox, Australia) spectrophotometer after 30 min.

The ferric reducing antioxidant power (FRAP) assay was accomplished as described by Benzie and Strain [86] with some modifications. The FRAP solution consisted TPTZ (0.01 M dissolved in 0.04 M HCl), FeCl_3_ × 6H_2_O (0.02 M in water), and acetate buffer (0.3 M, pH 3.6) at the ratio of 1:1:10. A volume of 2 mL of a recently prepared FRAP reagent was mixed with 2 μL of samples. The absorbance increase was established at 593 nm in a Cintra 202 (GBC Scientific Equipment, Knox, Australia) spectrophotometer after 30 min.

Calculation of all antioxidant activity assays was carried out using Trolox calibration curves and expressed as μmol of the Trolox equivalent (TE) per one gram of dry weight (µmol TE/g DW).

### 3.13. Evaluation of Color in Aralia cordata Different Motphological Parts

Color coordinates (L*, a*, b*) of samples powder were measured using the spectrophotometer MiniScan XE Plus (Hunter Associates Laboratory, Inc., Reston, Virginia, USA). CIEL*a*b* color coordinates were recorded as L* indicates the ratio of white to black color, value a*—the ratio of red (when a* > 0) to green (a* < 0) color, value b*—the ratio of yellow (b* > 0) to blue (b* < 0) color. Color saturation (the chroma value) was calculated (C = (a*^2^ + b*^2^)^1/2^), with a* and b* converted into hue angle (h° = arctan(b*/a*)) [87]. Before each analysis spectrophotometer is calibrated with a light catcher and standard white color, coordinates in color space are X = 81.3; Y = 86.2; Z = 92.7 of XYZ color. The color parameters were processed with the program “Universal Software V.4–10”. The measurements were established at least three times for each sample.

### 3.14. Scanning Electron Microscopy (SEM) Analysis

The morphology structure of the tested plant was examined from the images established by SEM FEI Quanta 200 FEG (FEI Company, Hillsboro, OR, USA). The *A. cordata* plant parts powder samples were spread on an aluminum table and measured at three different locations. The samples were analysed in a low vacuum mode operating at 3.0 kV using an LDF detector.

### 3.15. Statistical Analysis

The experiments were performed in triplicate, and all the results were expressed as mean value ± standard deviation. Principal Component Analysis (PCA) was performed with XLSTAT (Addinsoft, New York, NY, USA) on the abundances of the chemical composition and the different morphological parts of the plant to differentiate the samples and to analyze possible relationships between them. In addition, one-way ANOVA followed by Turkey’s HSD test to compare the means that indicate significant variation (*p* < 0.05) was accomplished and calculated with the statistical package GraphPad Prism 8 software (GraphPad, San Diego, CA, USA).

## 4. Conclusions

These results provide comprehensive and valuable biochemical data about *A. cordata* root, stem, leaf, inflorescence, berry, and seed. The highest anthocyanin and pro-anthocyanins content were detected in berries. However, the highest content of total chlorophyll and chlorophyll a and b were obtained in leaf. In addition, the most significant total phenolic content and antioxidant activity were investigated in the leaves, which are 2–3 times higher than other morphological parts. However, ascorbic acid content was highest in berries, reaching 146 mg/100 g, and covers the National Institutes of Health’s recommended dietary allowance by approximately 1.4 times for healthy adults. Moreover, plant parts powders showed an intensive color after freeze-drying and milling, which would be acceptable for an application in the food industry as a colorant. Additionally, it can be used as a safe material for green synthesis development as sugars and phenolics act as reducing and stabilizing agents. Moreover, the particle morphology of the obtained different *A. cordata* parts powders may affect the quality of the extracts and the antioxidant capacity of bioactive components in the food and pharmaceutical industry.

This study indicates that analyzed *A. cordata* morphological parts are a valuable source of phytochemicals and nutrients because of their antioxidant activity and bioactive compounds. Additionally, these results will provide the basis for the election of *A. cordata* plant morphological parts for further new botanical investigation for novel bioactive compounds and their application in broad industrial applications.

## Figures and Tables

**Table 1:** Composition of dry matter, invert sugar, sucrose, total sugars, and ascorbic acid contents of *A. cordata* different morphological parts.

Morphological Parts	Dry Matters, %	Invert Sugar, % DW	Sucrose, % DW	Total Sugars, % DW	Ascorbic Acid, mg/100 g DW
Root	92.4 ± 0.08 ^b^	4.8 ± 0.09 ^b^	0.8 ± 0.04 ^a^	5.7 ± 0.10 ^a^	22.3 ± 1.02 ^a^
Stem	97.6 ± 0.05 ^f^	6.9 ± 0.08 ^c^	2.6 ± 0.06 ^c^	9.6 ± 0.09 ^c^	66.4 ± 1.02 ^b^
Leaf	94.4 ± 0.11 ^d^	4.3 ± 0.06 ^a^	2.6 ± 0.08 ^c^	6.9 ± 0.05 ^b^	64.2 ± 0.47 ^b^
Inflorescence	93.3 ± 0.09 ^c^	10.2 ± 0.03 ^d^	1.3 ± 0.03 ^b^	11.5 ± 0.05 ^d^	67.2 ± 1.77 ^b^
Berry	90.9 ± 0.14 ^a^	44.2 ± 0.02 ^f^	6.5 ± 0.02 ^e^	50.8 ± 0.02 ^f^	146.0 ± 0.95 ^d^
Seed	96.0 ± 0.11 ^e^	24.1 ± 0.05 ^e^	4.5 ± 0.05 ^d^	28.6 ± 0.05 ^e^	76.8 ± 1.15 ^c^

Values expressed as a mean ± standard deviation (*n* = 3); different superscript letters within the same column indicate statistically significant differences (one-way ANOVA and Tukey’s HSD test, *p* < 0.05).

**Table 2:** The amount of chlorophyll contents of *A. cordata* morphological parts cited.

Morphological Parts	Total Chlorophyll Content, mg/g DW	Chlorophyll a Content, mg/g DW	Chlorophyll b Content, mg/g DW
Stem	0.22 ± 0.011 ^a^	0.05 ± 0.002 ^a^	0.26 ± 0.012 ^b^
Leaf	7.45 ± 0.071 ^c^	6.86 ± 0.061 ^c^	0.59 ± 0.024 ^c^
Inflorescence	0.59 ± 0.023 ^b^	0.50 ± 0.017 ^b^	0.08 ± 0.003 ^a^

Values expressed as a mean ± standard deviation (*n* = 3); the different superscript letters within the same column indicate statistically significant differences (one-way ANOVA and Tukey’s HSD test, *p* < 0.05).

**Table 3:** The total amount of phenolics, anthocyanins, and proanthocyanidins content of *A. cordata* morphological parts.

Morphological Parts	Phenolics, mg/100 g DW	Anthocyanins, mg/100 g DW	Proanthocyanidins, mg/100 g DW
Root	673.01 ± 12.147 ^b^	nd	nd
Stem	333.79 ± 9.905 ^a^	20.34 ± 0.813 ^b^	0.03 ± 0.001 ^a^
Leaf	2328.31 ± 47.269 ^e^	nd	0.06 ± 0.002 ^b^
Inflorescence	1084.18 ± 36.937 ^d^	nd	0.05 ± 0.002 ^ab^
Berry	913.21 ± 38.588 ^c^	78.54 ± 1.124 ^c^	0.28 ± 0.011 ^d^
Seed	356.42 ± 4.521 ^a^	18.27 ± 0.303 ^a^	0.10 ± 0.001 ^c^

nd: not detected. Values expressed as a mean ± standard deviation(*n* = 3); different superscript letters within the same column indicate statistically significant differences (one-way ANOVA and Tukey’s HSD test, *p* < 0.05).

**Table 4:** The total and individual carotenoid content (mg/g DW) of *A. cordata* morphological parts.

Morphological Parts	Carotenoids, mg/g DW	Lutein, mg/g DW	Zeaxanthin, mg/g DW	β-Carotene, mg/g DW
Stem	0.244 ± 0.0122 ^b^	0.128 ± 0.0065 ^b^	0.081 ± 0.0021 ^b^	0.035 ± 0.0016 ^c^
Leaf	0.812 ± 0.0406 ^d^	0.291 ± 0.0047 ^d^	0.195 ± 0.0051 ^d^	0.221 ± 0.0075 ^e^
Inflorescence	0.420 ± 0.0187 ^c^	0.271 ± 0.0122 ^c^	0.110 ± 0.0029 ^c^	0.024 ± 0.0006 ^b^
Berry	0.115 ± 0.0032 ^a^	0.012 ± 0.0004 ^a^	nd	0.071 ± 0.0032 ^d^
Seed	0.035 ± 0.0015 ^a^	0.011 ± 0.0004 ^a^	0.010 ± 0.0003 ^a^	0.013 ± 0.0005 ^a^

nd: not detected. Values expressed as a mean ± standard deviation (*n* = 3); different superscript letters within the same column indicate statistically significant differences (one-way ANOVA and Tukey’s HSD test, *p* < 0.05).

**Table 5:** The results of color analysis characteristic of *A. cordata* morphological parts.

Morphological Parts	Color Parameters				
	L*	a*	b*	C	h°
Root	70.2 ± 0.01 ^e^	3.00 ± 0.067 ^d^	17.9 ± 0.15 ^e^	18.2 ± 0.14 ^d^	80.5 ± 0.28 ^c^
Stem	72.1 ± 0.08 ^f^	−2.13 ± 0.015 ^c^	14.4 ± 0.12 ^c^	14.5 ± 0.12 ^c^	98.4 ± 0.14 ^d^
Leaf	54.5 ± 0.04 ^c^	−6.64 ± 0.086 ^a^	16.4 ± 0.08 ^d^	17.7 ± 0.06 ^d^	112.1 ± 0.32 ^e^
Inflorescence	64.1 ± 0.01 ^d^	−3.41 ± 0.045 ^b^	21.7 ± 0.07 ^f^	22.0 ± 0.07 ^f^	98.9 ± 0.10 ^d^
Berry	36.7 ± 0.03 ^a^	5.29 ± 0.125 ^e^	3.5 ± 0.07 ^a^	6.4 ± 0.07 ^a^	33.8 ± 1.12 ^a^
Seed	39.7 ± 0.06 ^b^	5.98 ± 0.105 ^f^	7.0 ± 0.12 ^b^	9.22 ± 0.12 ^b^	49.6 ± 0.63 ^b^

Color coordinate L* indicates brightness value, a* coordinate indicates greeness (negative value) or redness (positive value), b* coordinate indicates blueness (negative value) or yellowness (positive value), C value indicates chroma (color purity or relative saturation), and h° value indicates angle of the hue in the CIELAB color space. Values are expressed as a mean ± standard deviation (*n* = 3); different superscript letters within the same column indicate statistically significant differences (one-way ANOVA and Tukey’s HSD test, *p* < 0.05).

## Data Availability

All data generated during this study are included in this article.
